# Inter- and intra-observer reproducibility of ADC measurements in esophageal carcinoma primary tumors

**DOI:** 10.18632/oncotarget.21639

**Published:** 2017-10-06

**Authors:** Zhimin Ye, Jun Fang, Shujun Dai, Tieming Xie, Fangzheng Wang, Zhun Wang, Kai Li, Zhenfu Fu, Yuezhen Wang

**Affiliations:** ^1^ Department of Radiation Oncology, Zhejiang Cancer Hospital, Hangzhou, China; ^2^ Department of Intensive Care Unit, The Second Affiliated Hospital, Zhejiang University School of Medicine, Hangzhou, China; ^3^ Department of Radiology, Zhejiang Cancer Hospital, Hangzhou, China

**Keywords:** esophageal carcinoma, diffuse weighted imaging, apparent diffused coefficient, reproducibility

## Abstract

The apparent diffuse coefficient (ADC) may correlate with the treatment response to chemotherapy/radiotherapy in solid tumors. Our aim was to determine the inter- and intra-observer reproducibility of ADC measurements in primary esophageal squamous cell carcinoma (ESCC). ADCs were blindly measured in 31 patients diagnosed with ESCC by two observers before treatment (pre-ADC) and after 5^th^ fraction radiotherapy (intra-ADC) twice with a 2-week interval. The mean pre-ADC of primary tumors was 1.25±0.22 and 1.27±0.23 (in 10^−3^mm^2^/s) from observer A for measurements 1 and 2, respectively, and the intra-observer measurements were -0.02 bias vs. -0.13-0.09 limits of agreement. From observer B, the mean pre-ADC varied between 1.25±0.23 and 1.27±0.23 (in 10^−3^mm^2^/s) for measurements 1 and 2, respectively, and intra-observer measurements were -0.02 bias vs. -0.17∼0.16 limits of agreement. The mean pre-ADC of primary tumors was 1.26±0.24 (in 10^−3^mm^2^/s) from observers A and B, and inter-observer measurements were 0.01 bias vs. -0.09-0.09 limits of agreement, revealing a low inter-observer variance. Similar measurements of the intra-SD parameters showed that the pre- and intra-ADC of primary tumors differed significantly. Thus ADC measurements may have sufficient inter-observer and intra-observer reproducibility to measure primary tumor responses to treatment, and the ADCs before and during treatment differed.

## INTRODUCTION

Treatment response of esophageal carcinoma (EC) is affected by many factors including oxygenation status of cancer cells, gene mutation, and distribution of microvascular vessels [1–3]. There is also variability in radiotherapy dosage among different treatment centers. For example, the RTOG 94-05 phase III trials demonstrated that the survival or local/regional control in the group of higher radiation dose (64, 8Gy) was not increased compared with that in the group of lower radiation dose (50.4Gy) [4]. Moreover, in the CROSS phase III trial, disease-free survival (DFS) and overall survival were improved in patients underwent radiation doses of 41.4Gy preoperative chemoradiotherapy (CRT) compared to patients that underwent surgery alone [5]. If the sensitivity of chemotherapy/radiotherapy response is monitored early, the effectiveness of these treatment regimens will be better predicted.

Diffusion-weighted imaging (DWI) is a functional approach that detects water molecule diffusion in the body, and the apparent diffusion coefficient (ADC) has been utilized for the clinical application of evaluating the treatment response to CRT in many cancers [6]. Clinically valid use of DWI requires that measurement variation in a given patient be less than that observed by different observers or measurement. The inter- and intra-reproducibility of ADC measurement is rarely reported for primary tumors of EC patients, and contouring of the measurement region of interest (ROI) is not standardized. The variation of protocols, b-values, and calculations reported by different institutions to obtain the ADC values and cut-off values cannot be compared and are not possible to utilize clinically [7, 8]. Therefore, the broad application of DWI in the prediction of treatment response is dependent on the accuracy and reproducibility of the measurements.

We estimated the reproducibility of two measurements of ADC (at baseline and the 5^th^ fraction of RT) via a designated method and explored the change in ADC during the early stages of treatment.

## RESULTS

### General clinical data of 31 patients

A total of 31 patients (20 men, 11 women; mean age 64.5±8.7 years) were diagnosed with esophageal squamous cell carcinoma (ESCC). The number of patients in each T stage were T1, n = 0, T2, n = 3, T3, n = 24, T4, n = 4, respectively. Three cases scored performance status (PS) of 0, 16 cases scored PS of 1, and 12 cases scored PS of 2. Primary tumor sites were located in the neck of 3 cases, upper thoracic of 4 cases, middle thoracic of 17 cases, and lower thoracic of 7 cases. The mean RT dose was 5800.65±647.94 cGy.

### Measurement and reproducibility of ADC and SD before treatment

**Figure 1:**
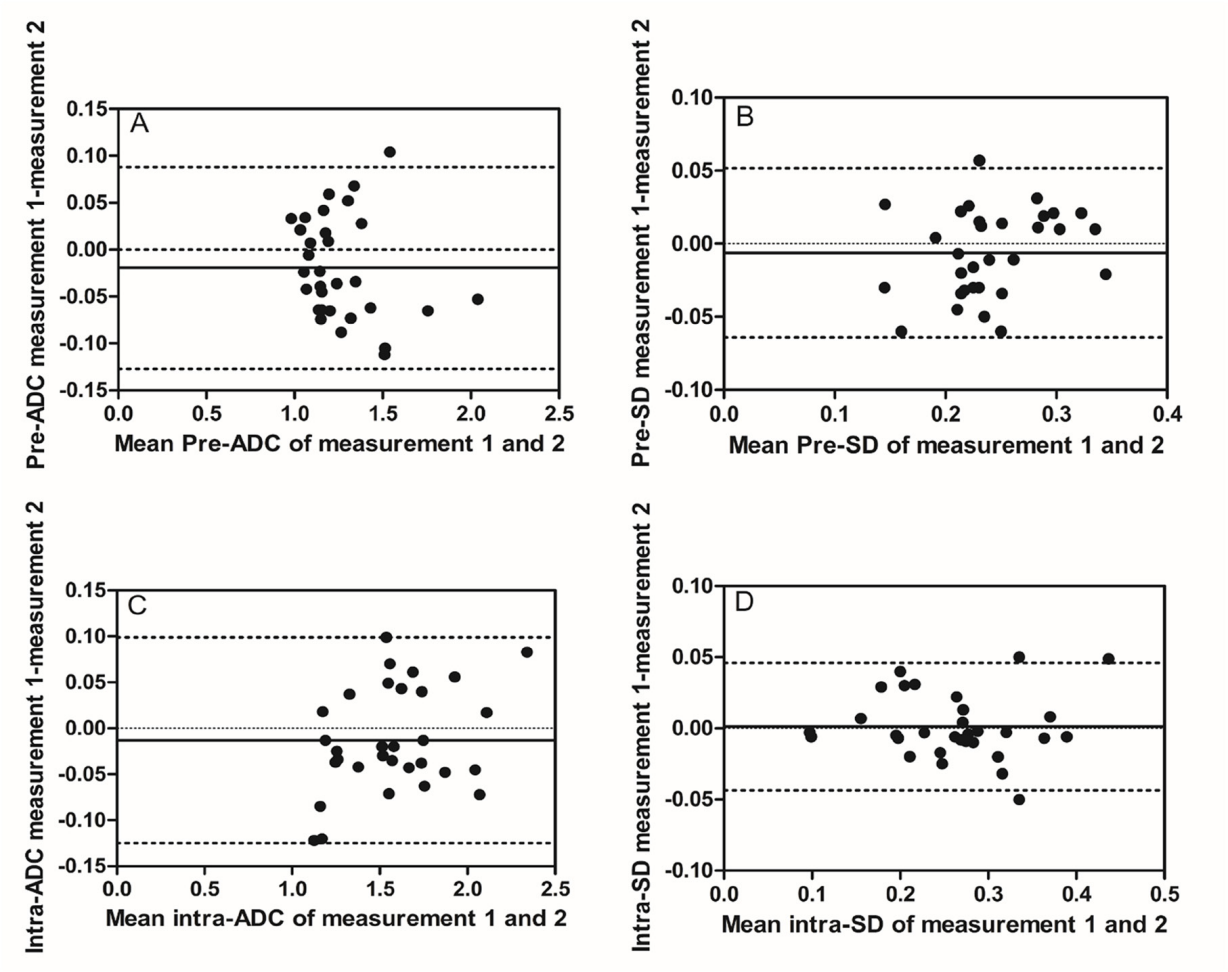
Intra-observer reproducibility of primary tumor ADC and SD measurements from observer A Bland–Altman plots of difference of ADC or SD measurements (y-axis) vs. mean ADC or SD measurement (x-axis), with mean absolute difference (bias) (continuous line) and 95% confidence interval (CI) of the mean difference (limits of agreement) (dashed lines except zero line). **(A)** The measurement of pre-ADC in primary tumor, **(B)** the measurement of pre-SD in primary tumors, **(C)** the measurement of intra-ADC in primary tumors, and **(D)** the measurement of intra-SD in primary tumors. The results showed relatively good intra-observer reproducibility with most plots distributed within the 95%CI.

Before treatment, mean pre-ADC of primary tumors were 1.25±0.22 and 1.27±0.23 (in 10^−3^mm^2^/s) from observer A for the measurement 1 and 2 respectively, and 1.25±0.23, 1.27±0.23 from observer B, respectively (Table 2). The intra-observer measurements were -0.02 bias vs. -0.13∼0.09 limits of agreement from observer A and -0.02 vs. -0.17∼0.16, respectively, from observer B (Figure 1A, 2A). The mean pre-SD of primary tumors for measurement 1 and 2, respectively, were 0.24±0.05 and 0.25±0.05 (in 10^−3^mm^2^/s) from observer A and 0.22±0.05, 0.24±0.05, from observer B (Table 3). The intra-observer measurements were -0.01 bias vs. -0.06∼0.05 limits of agreement from observer A and -0.02 vs. -0.11∼0.08, respectively from observer B (Figure 1B, 2B). The inter-observer measurements in pre-ADC and pre-SD were 0.01 bias vs. -0.09∼0.10 limits of agreement and 0.01 vs. -0.07∼0.09, respectively (Figure 3A, 3C).

**Table 1:** General clinical characteristics of study population (n=31)

Character	No
Gender	
M	20
F	11
Age (years)	64.5±8.7
PS	
0	3
1	16
2	12
T stage	
T1	0
T2	3
T3	24
T4	4
Location of tumor	
Neck	3
Upper thoracic	4
Middle thoracic	17
Lower thoracic	7
Chemotherapy	
Neo-adjuvant	20
CCRT	24
Dose of RT (cGy)	5800.65±647.94

**Table 2:** Twice measurements of pre-ADC in observer A and B

Observer	Measurement	Mean pre-ADC (in 10^−3^mm^2^/s)
A	1	1.25±0.22
	2	1.27±0.23
B	1	1.25±0.23
	2	1.27±0.23

**Figure 2:**
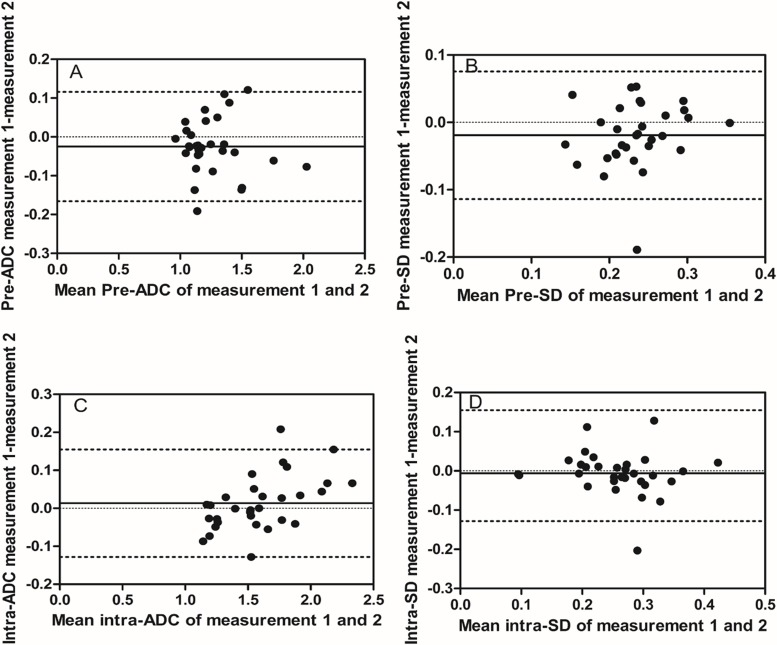
Intra-observer reproducibility of primary tumor ADC and SD measurements from observer B Bland–Altman plots of difference of ADC or SD measurements (y-axis) vs. mean ADC or SD measurement (x-axis), with mean absolute difference (bias) (continuous line) and 95% confidence interval of the mean difference (limits of agreement) (dashed lines except zero line). **(A)** The measurement of pre-ADC in primary tumors, **(B)** the measurement of pre-SD in primary tumors, **(C)** the measurement of intra-ADC in primary tumors, and **(D)** the measurement of intra-SD in primary tumors.

**Table 3:** Twice measurements of pre-SD in observer A and B

Observer	Measurement	Mean pre-SD (in 10^−3^mm^2^/s)
A	1	0.24±0.05
	2	0.25±0.05
B	1	0.22±0.05
	2	0.24±0.05

**Figure 3:**
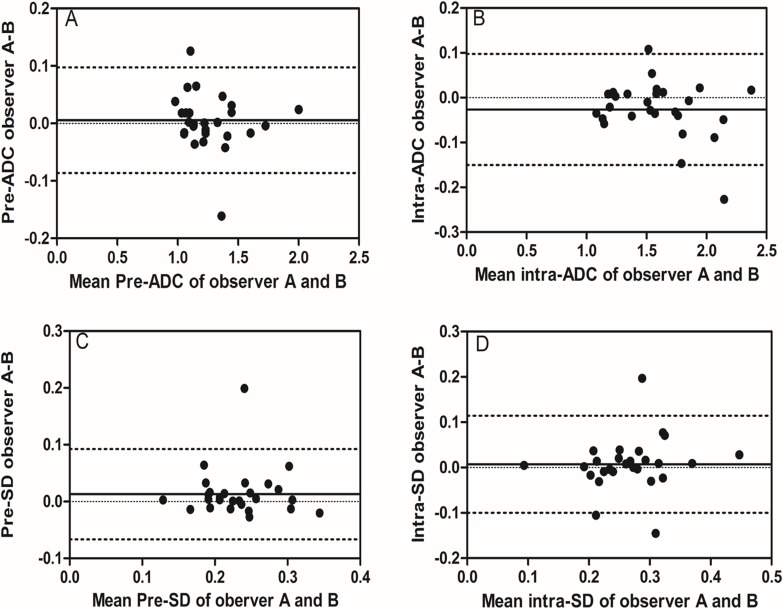
Inter-observer reproducibility of DWI primary tumor ADC measurements from observer A and B Bland–Altman plots of difference of ADC or SD measurements (y-axis) vs. mean ADC measurement (x-axis), with mean absolute difference (bias) (continuous line) and 95% confidence interval (CI) of the mean difference (limits of agreement) (dashed lines except zero line). The results showed that inter-observer reproducibility was acceptable, which displayed most plots distributed between the lines of 95% CI. **(A)** The measurement of pre-ADC between observer A and B, **(B)** the measurement of intra-ADC between observer A and B, **(C)** the measurement of pre-SD between observer A and B, and **(D)** the measurement of intra-SD between observer A and B.

### Measurement and reproducibility of ADC and SD during treatment

At the 5^th^ RT, the mean intra-ADC of primary tumors from observer A was 1.57±0.32 and 1.59±0.30 (in 10^−3^mm^2^/s) for measurement 1 and 2 respectively, and 1.60±0.34, 1.58±0.33 from observer B (Table 4). The bias vs. limits of agreement for the intra-observer measurements corresponding to Bland–Altman plots from observer A and B are displayed in Figure 1C, 2C, respectively. The inter-observer measurements of intra-ADC were -0.03 bias vs. -0.15∼0.10 limits of agreement (Figure 3B). The mean intra-SD of primary tumors from observer A and B is summarized in Table 5 for measurement 1 and 2, and the intra-observer bias vs. limits of agreement are displayed in Figure 1D, 2D. The inter-observer bias vs. limits of agreement for the intra-ADC and intra-SD are shown in Figure 3B, 3D, respectively.

**Table 4:** Twice measurements of intra-ADC in observer A and B

Observer	Measurement	Mean intra-ADC (in 10^−3^mm^2^/s)
A	1	1.57±0.32
	2	1.59±0.30
B	1	1.60±0.34
	2	1.58±0.33

**Table 5:** Twice measurements of intra-SD in observer A and B

Observer	Measurement	Mean intra-SD (in 10^−3^mm^2^/s)
A	1	0.26±0.08
	2	0.28±0.07
B	1	0.25±0.07
	2	0.26±0.07

### Differential analysis of ADC and SD measurements

**Figure 4:**
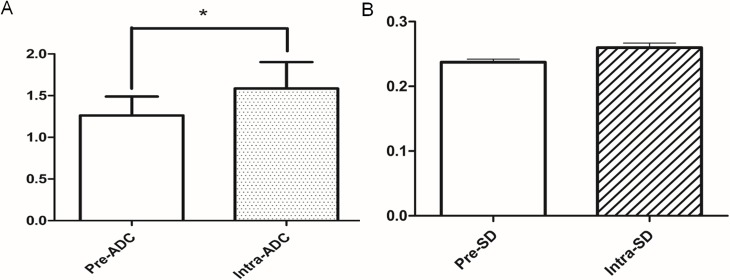
The comparison of ADC and SD parameters between pre-treatment and the 5^th^ RT **(A)** The value of intra-ADC was higher than that of pre-ADC (^*^:P<0.05); **(B)** the value of intra-SD was higher than that of pre-SD, but the difference was not statistically significant (P>0.05).

Compared to the value of pre-ADC, the value of intra-ADC was significantly higher (P<0.05, Figure 4A). However, while the value of intra-SD was higher than pre-SD, there is no significant difference between pre-SD and intra-SD (P>0.05, Figure 4B).

## DISCUSSION

Functional imaging such as DWI is increasingly prominent in the treatment response evaluation of esophageal carcinoma due to the recent widespread application of MR for esophagus examination. However, a major challenge to the interpretation of functional metabolic imaging-generated parameters, including the ADC value of DWI, is the inherent physiologic heterogeneity within a tumor. To our best of knowledge, there is no standard protocol for performing ADC measurements of esophageal carcinoma.

Notably, few published studies have investigated the clinical value of DWI in evaluating esophageal carcinoma. Several studies have used “whole tumor” ROI data to differentiate malignant and benign nodes in esophageal carcinoma [10, 11] and predict RT response [12, 13]. Some groups have advocated assessment of only the most enhanced voxels within a tumor, based on the result that the most enhanced ROIs provided more statistically significant differences between responders and non-responders in CRT than whole tumor ROI [14]. Many studies neglect to illustrate the delineation of ROI and do not report intra- and inter-observer reproducibility of the ADC measurement [15, 16].

**Figure 5:**
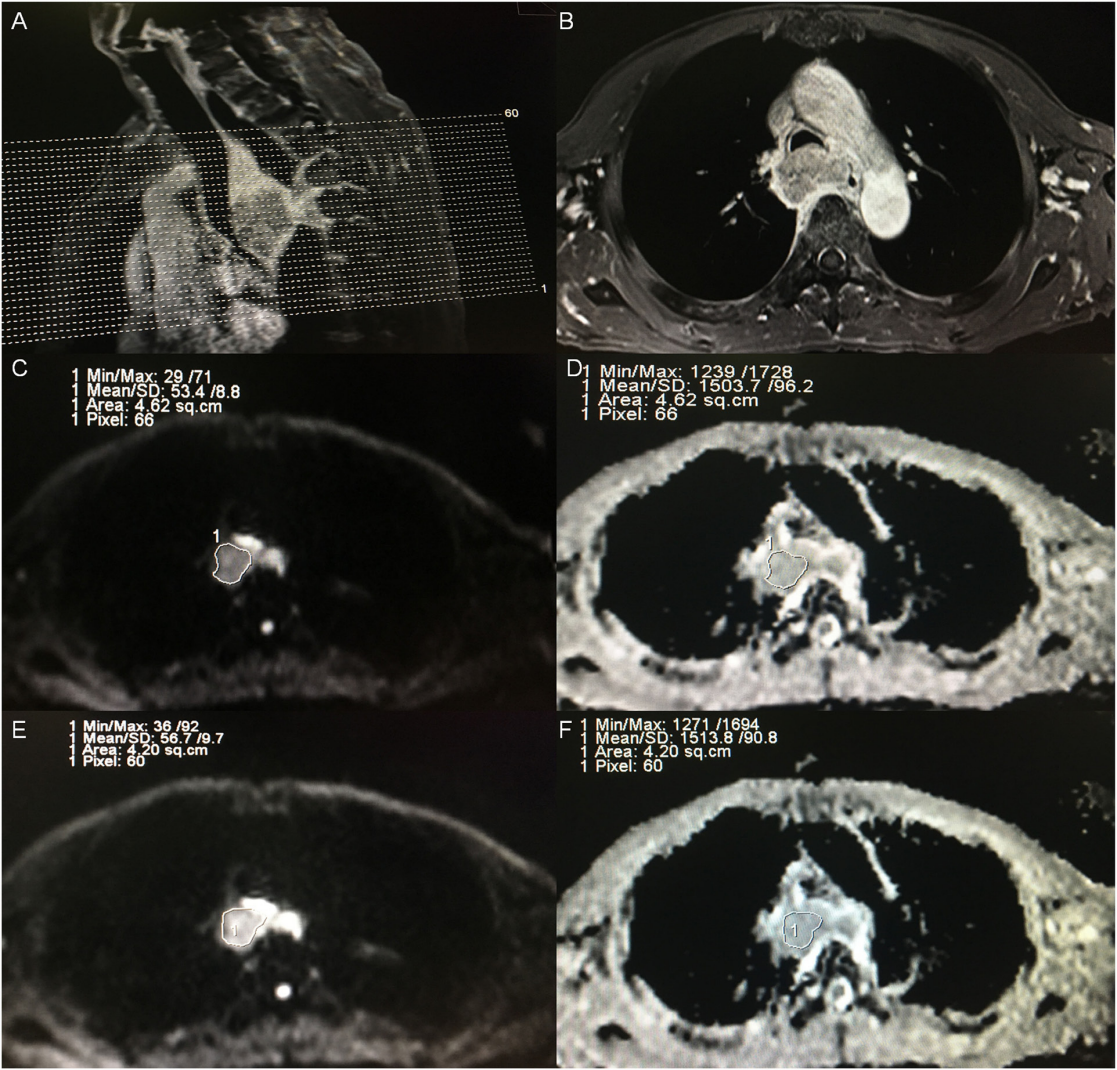
Example of ADC measurements of a primary tumor in a patient diagnosed as esophageal carcinoma before treatment **(A)** A sagittal view with position line of T1-weighted plus contrast-enhanced (T1+C) image for the primary tumor. **(B)** A transverse view of T1+C image for the one of three continuous sections with maximal diameter of tumor according to the sagittal and transverse view. **(C)** A region of interest (ROI) was placed manually for observer A in the selected section, on the image obtained at a b-value of 1000 s/mm^2^, and the ROI was then copied and pasted onto the ADC map **(D)**, and the ADC and SD of the selected section were automatically calculated. **(E)** A region of interest (ROI) was placed manually for observer B in the selected section, on the image obtained at a b-value of 1000 s/mm^2^, and the ROI was then copied and pasted onto the ADC map **(F)**, and the ADC and SD were also automatically calculated.

Our study specifically addressed ROI selection strategies to estimate intra- and inter-observer reproducibility. The method of ROI contouring in our study relied on the following strategies: (1) ROI in the slice containing the most enhanced voxels in enhanced contrast T1WI and excluded the necrotic areas to avoid intra-tumoral variation [9, 17, 18], and the point was widely recommended for the measurement of ADC; and (2) Selection of three continuous sections, including the largest slice, to determine the average ADC of the tumor (Figure 5). Our method was derived from previous studies [19, 20] where the delineation was based on the largest slice, but the ROI our study was not confined to the largest slice to assure low variance during the period of ROI delineation. Our data suggest that this is an appropriate strategy to assure the reproducibility of intra-observer and inter-observer. Furthermore, the resulting bias and limits of agreement measurements were acceptable, and low variance in ADC measurements was indicated by the parameter SD. Our results were consistent with Kwee et al. [21] who determined that semi-automated volumetric ADC measurements were more reproducible than manual ADC measurementxoldaxas. However, Kwee et al. [22] revealed that despite good inter- and intra-observer reproducibilities, the ADC value was not always sufficiently reproducible to discriminate malignant from non-malignant lymph nodes.

Interestingly, we also found that the ADC was significantly different at 5^th^ fraction RT which showed the change of functional parameters preceded the change of anatomical morphology. This result suggests the potential of ADC to predict the treatment response of esophageal carcinoma earlier. The check-point of monitoring response may shift earlier to avoid interference from tumor reduction that causes measurement error. The optimal check-point of treatment response is still controversial [8, 14, 15], so the method in our study may be an alternative to monitoring early treatment response. Studies are ongoing in our center.

In conclusion, the ADC measurement from DWI is highly reproducible in esophageal carcinoma via our method and could predict treatment response.

## MATERIALS AND METHODS

### Patient selection

Thirty-one patients (20 men, 11 women; mean age 64.5±8.7 years; age range 41–79 years) diagnosed with ESCC by pathology, and treated at Zhejiang provincial cancer hospital between January 2015 and November 2016 were enrolled in this study. The study was approved by the institutional review board, and written informed consent was obtained from each participant before MRI examination. All subjects were qualified by the following criteria: (1) Eastern Cooperative Oncology Group performance status score is smaller than or equal to 2; (2) Adequate organ function; (3) No concomitant malignancy; (4) Good compliance; (5) No contraindication to MRI examination; (6) No surgical indications or patient refusal; (7) and completion of the entire course of radiotherapy. The stage of disease was classified according to the 7th edition of the Union for International Cancer Control (UICC) and the American Joint Committee on Cancer (AJCC) staging system. The clinical characteristics of all patients are listed in Table 1.

### MR imaging

All subjects underwent two MRI examinations with a 1.5-T MR scanner (Achieva, Philips Medical Systems, Best, The Netherlands) using a phased array body coil (SENSE body coil, Philips Medical Systems, Best, The Netherlands). Examinations were performed before the initiation of treatment and at the 5^th^ fraction radiotherapy (RT) point. All MRI examinations contained axial spin-echo T1 weighted imaging (TR/TE 423/100 ms, average number 1, FOV 365×284mm, matrix 320, slice thickness 4 mm, skip 1.2–1.6 mm and slice 20), axial turbo spin-echo T2 weighted imaging (TR/TE 2,000/70 ms, flip angle 180°, concatenations 2, average number 2, FOV 300×280 mm, matrix 288, slice thickness 4 mm, skip 1.2–1.6 mm and slice 20), T1 with contrast enhanced imaging including sagittal and transverse axial, and then DWI (TR/TE/TI 10,205/70/180 ms, FOV 450×366mm, matrix 256, slice thickness/gap 4/0 mm, slice 20, EPI factor (echo train length) 43). DWI scans were obtained using a single-shot spin-echo type of echo-planar sequence, and fat signals were suppressed using short-tau inversion recovery (STIR). The DWI b-values were b=0 and 1,000s/mm^2^. An interval of 7 min was allocated to acquire DWI with free breathing.

### Imaging analysis

Both MR images were transferred to a workstation (ViewForum; Philips Medical Systems, Best, The Netherlands). Two board-certified radiologists (observer 1, Tieming Xie, with 13 years of experience in MR imaging; observer 2, Mingxiang Jiang, with 12 years of experience in MR imaging) reviewed the images and recorded the locations and slice numbers of the primary tumor site independently and blindly, and then performed ADC measurements of the selected tumor through the contouring region of interest (ROI). Each ROI was variable so that the two observers obeyed the following stipulations: (1) Used ROI in the slice containing the most enhancing voxels in enhanced contrast T1WI [9]. (2) Avoided the non-enhancement or necrotic areas in the ROIs. (3) Selected three continuous slices including the one of maximal diameter and its adjacent above and below one in tumor parenchyma according to the sagittal and horizontal view, and the values of ADC were averaged based on the three slices. (4) All measurements were performed twice by each observer, with a wash-out period of at least two weeks between the first and second series of measurements. The pre-treatment and 5^th^ RT ADCs were labeled as pre-ADC and intra-ADC, respectively.

### Statistical analysis

The mean ADC±SD of primary tumors including pre-ADC, pre-SD, intra-ADC, and intra-SD were acquired by each observer for each series of measurements. Secondly, inter- and intra-observer reproducibility of primary tumor ADC measurements tumor was determined by mean absolute difference (bias) and 95% confidence interval of the mean difference (limits of agreement) according to the methods of Bland and Altman. Bland-Altman plots were constructed by GraphPad-Prism 5 software. Statistical analyses were executed using SPSS 16.0 software (SPSS, Chicago, IL, USA), and the analysis of variance (ANOVA) was performed to compare the continuous variables between two groups.

## Abbreviations

ADC: apparent diffused coefficient
DWI: diffusion-weighted imaging
RT: radiotherapy
ROI: region of interest
ESCC: esophageal squamous cell carcinoma
PS: performance status.
